# A Comparison of the Clinical and Epidemiological Profile of Rocky Mountain Spotted Fever with Dengue and COVID-19 in Hospitalized Children, Sonora, México, 2015–2022

**DOI:** 10.3390/tropicalmed10010020

**Published:** 2025-01-14

**Authors:** Gerardo Álvarez-Hernández, Cristian Noé Rivera-Rosas, Jesús René Tadeo Calleja-López, Jehan Bonizú Álvarez-Meza, Maria del Carmen Candia-Plata, Denica Cruz-Loustaunau, Antonio Alvídrez-Labrado

**Affiliations:** 1 Department of Medicine and Health Sciences, University of Sonora, Blvd. Luis D. Colosio SN, col. Centro, Hermosillo 83000, Mexico; carmen.candia@unison.mx; 2 Epidemiological Surveillance Unit, Family Medicine Unit 53, Mexican Institute of Social Security, Zapopan 45100, Mexico; md.cristian.rivera@gmail.com; 3 Internal Medicine Service, General Zone Hospital 14, Mexican Institute of Social Security, Hermosillo 83260, Mexico; jrtcalleja@gmail.com; 4 Children’s Hospital of the State of Sonora, Hermosillo 83100, Mexico; bonizu1501@hotmail.com (J.B.Á.-M.); denica_cruz@hotmail.com (D.C.-L.); 5 Department of Medicine and Health Sciences, University of Sonora, Ciudad Obregon 85199, Mexico; antonio.alvidrez@unison.mx

**Keywords:** Rocky Mountain spotted fever, dengue, COVID-19, *Rickettsia rickettsii*, vector-borne diseases, coronavirus, SARS-CoV-2

## Abstract

Background: Rocky Mountain spotted fever (RMSF) is a challenge for physicians because the disease can mimic other endemic febrile illnesses, such as dengue and COVID-19. The comparison of their main clinical and epidemiological manifestations in hospitalized children can help identify characteristics that improve empirical suspicion and timely therapeutic interventions. Methods: A cross-sectional study was conducted on a series of patients aged 0 to 18 years, hospitalized between 2015 and 2022, with a diagnosis of RMSF, dengue, or COVID-19. Data were retrieved from medical records. Subjects were categorized as patients with RMSF (group I) and patients with dengue and COVID-19 (group II). Descriptive statistics were used, and differences were evaluated using Student’s t-test and the chi-squared test. Results: A series of 305 subjects were studied, with 252 (82.6%) in group I. Subjects in both groups presented fever, myalgias, arthralgias, and rash, but exposure to ticks distinguished group I. The fatality rate (21.0%) in group I was higher than in group II (3.8%). Conclusions: Although fever, myalgias, arthralgias, and rash are common in all three illnesses, they are more prevalent in hospitalized patients with RMSF. In the presence of such symptoms, a history of tick exposure can guide clinical decisions in regions where all three diseases are endemic.

## 1. Introduction

Rocky Mountain spotted fever (RMSF) is a potentially lethal zoonotic disease, caused by the obligated intracellular Gram-negative bacteria *Rickettsia rickettsii*, which is transmitted to hosts by Ixodidae ticks of the *Dermacentor*, *Amblyomma*, and *Rhipicephalus* genera [1,2,3]. RMSF is geographically distributed in countries of the Americas, primarily in Brazil [4], México, and the United States [5,6], although it has also been contemporaneously documented in other countries such as Panama [7], Costa Rica [8], Argentina, and Colombia [9,10].

In Mexico, RMSF is a public health problem, mostly in the northern region of the country, where vulnerable populations, particularly children < 10 years old, primarily if they live in social disadvantage, suffer the highest impact of this disease. In this group, case fatality rates (CFRs) ranging between 27% and 58% have been registered in Mexican hospitalized patients [11,12]. Delayed initiation with doxycycline, a specific antibiotic, is the main factor associated with this CFR [6,13], which is mostly related to the community and physicians’ knowledge about the clinical picture of the disease and how to distinguish it from other febrile regionally endemic diseases such as dengue and, recently, COVID-19 [5,14].

The lack of early specific clinical features of RMSF challenges clinicians to differentiate it from other febrile rashes, delaying early suspicion and appropriate drug management [15]. At least for the two last decades, RMSF and dengue presented cases and outbreaks every year in Sonora, located in the northwest region in Mexico [16], while the COVID-19 pandemic provoked 16,215 cases in pediatric patients from the same region during 2020–2023 [17]. Overall, these three diseases display some similar pathophysiological mechanisms underlying a proinflammatory, procoagulant, and immune state. These processes result in endothelial vascular damage that explains most of the symptoms and medical complications observed in these diseases [18,19,20].

By comparing clinical and epidemiological features of RMSF, dengue, and COVID-19 in pediatric patients, this study can contribute to guide medical practice for an early suspicion and specific treatment in regions where all three diseases are endemic.

## 2. Materials and Methods

### 2.1. Study Design

**Figure 1:**
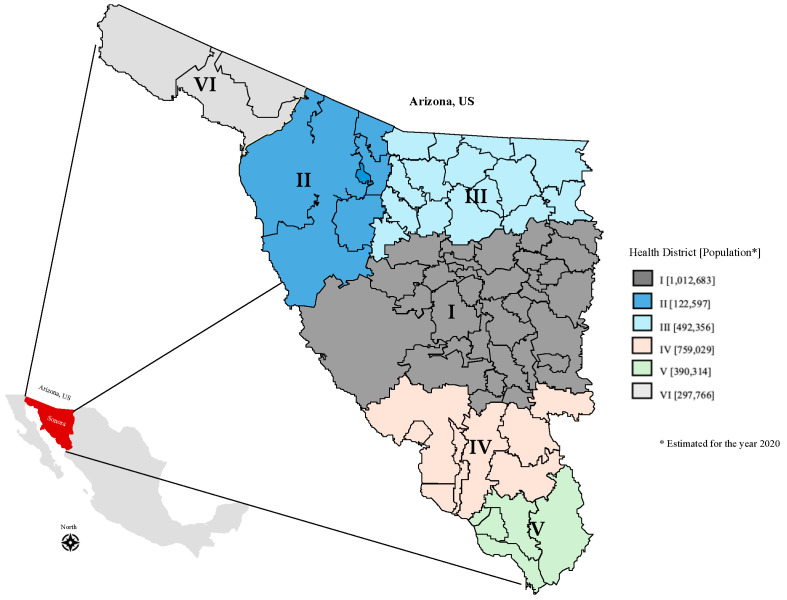
Health districts in Sonora, Mexico, 2024.

A retrospective cross-sectional study was conducted in a consecutive case series of pediatric patients aged between 0 and 18 years with the diagnosis of Rocky Mountain spotted fever, using the diagnosis codes of spotted fever due to *Rickettsia rickettsii* and Rickettsiosis unspecified (A77.0 and A77.9); dengue without warning signs and dengue with warning signs (A90.0 and A91.0); or COVID-19 (U07.1), according to the International Classification of Diseases (ICD-10) [21,22]. The subjects were from different health districts of Sonora (Figure 1) admitted in the Children’s Hospital of the State of Sonora between 1 January 2015 and 31 December 2022. All the study procedures were approved by the Research Ethics Committee from the study site.

### 2.2. RMSF Diagnosis

The diagnosis of RMSF was established in each patient who presented an acute clinical onset (less than one week) with fever, headache, malaise, and rash, accompanied either by the following: (1) The detection of *R. rickettsii* or *Rickettsia* spp. in a single blood sample using real-time polymerase chain reaction (RT-PCR). The *Rickettsia* PCR amplification of the gltA gene was made through the Taqman Gene Expression Master Mix, Applied Biosystems^®^ catalog 4369016 (Thermo Scientific, Waltham, MA, USA), while the species was identified by the hypothetical protein A1G_04230, with an amplification product of 153 base pairs [23]. The rickettsial DNA was extracted by using either the commercial kits QIAamp™ DNA Blood Kits, the QIAam™ DNA Mini Kit (QIAGEN, Mexico City, Mexico), or the MagNA Pure 24 System™ (Roche Diagnostics, Mexico City, Mexico). (2) The serum determination of IgG titles ≥ 1:64 of *R. rickettsii* antibodies in a single blood sample through an indirect immunofluorescence assay (IFA). The slides for this technique were prepared by the National Institute for Epidemiological Diagnosis and Reference (InDRE), and such slides contain specific antigens for *R. rickettsii*. (3) Clinical and epidemiological criteria, as judged by the clinicians in charge, even in the absence of a positive laboratory result either by RT-PCR or IFA.

### 2.3. Dengue Diagnosis

Dengue was present in a patient with febrile illness confirmed by the detection of dengue virus using RT-PCR in a single serum sample drawn at hospital admission. The detection of dengue virus was made by using the commercial kit TaqMan™ Arbovirus Triplex Kit (ZIKV/DENV/CHIKV), 0.1 mL (Thermo Scientific, Waltham, MA, USA).

### 2.4. COVID-19 Diagnosis

Regarding COVID-19, a patient must present a febrile illness and have a positive result for the SARS-CoV-2 virus in a sample taken from a nasopharyngeal swab processed either by RT-PCR (TaqPath™ COVID-19, FluA, FluB Combo Kit) (Thermo Scientific, Waltham, MA, USA) or through the positive antigen of SARS-CoV-2.

All the procedures to confirm the diseases were performed in the Sonora State Public Health Laboratory, the reference laboratory for diseases under epidemiological surveillance.

### 2.5. Statistical Analysis

The sampling frame was based on 427 medical records with the aforementioned discharge diagnoses from the study period. The subjects were included if they fulfilled the following criteria: (1) less than 18 years old, (2) if they received medical attention at any service of the hospital, (3) and if they resided in Sonora. Patients excluded were those with documented comorbidities in their medical records (i.e., cancer, autoimmune diseases, viral hepatitis, or HIV). The final study sample constituted of 305 patients. Clinical and epidemiological data were retrieved from medical charts for the study sample, which was classified in two groups for comparison purposes. Group I constituted of patients with RMSF diagnosis, whereas group II included patients with dengue or COVID-19.

All data on clinical, laboratory, and epidemiological characteristics were collected at hospital admission. This information was captured into a database by two clinicians who were previously trained for data collection but were not blinded to the study purposes.

Descriptive statistical analysis was conducted; relative frequencies tables and graphics were created to display the results. Differences between both groups were analyzed through the t-test, Mann–Whitney test, and chi-squared test as required; *p* < 0.05 values were considered statistically significant. To examine differences in the risk distribution of selected clinical variables, risk ratios and their confidence interval at 95% were estimated. The statistical analysis was performed using the Number Cruncher Statistical System (NCSS) 2023 version 23.0.2.

## 3. Results

Our study sample constituted of 305 patients; 252 (82.6%) of them corresponded to group I (RMSF). One hundred and fifty (59.5%) patients within this group received a laboratory confirmation, while in group II, all of the patients had a confirmatory laboratory test (*p* < 0.001). There were no statistical differences regarding sex distribution (*p* = 0.1780) and the mean age of patients [group I: 8.86 ± 4.29, group II: 8.21 ± 6.55] (*p* = 0.489); however, it was observed that 60.0% of the RMSF patients were younger than 10 years old, while in group II, 52.9% were above 10 years (*p* ≤ 0.001). With regard to epidemiological characteristics, group I showed higher proportions of patients living in low socioeconomic conditions (79% vs. 62.2%), residing in urban areas (90.5% vs. 81.1%), and having a history of tick exposure (92.1% vs. 9.4%), and all of these differences were statistically significant (Table 1).

**Figure 2:**
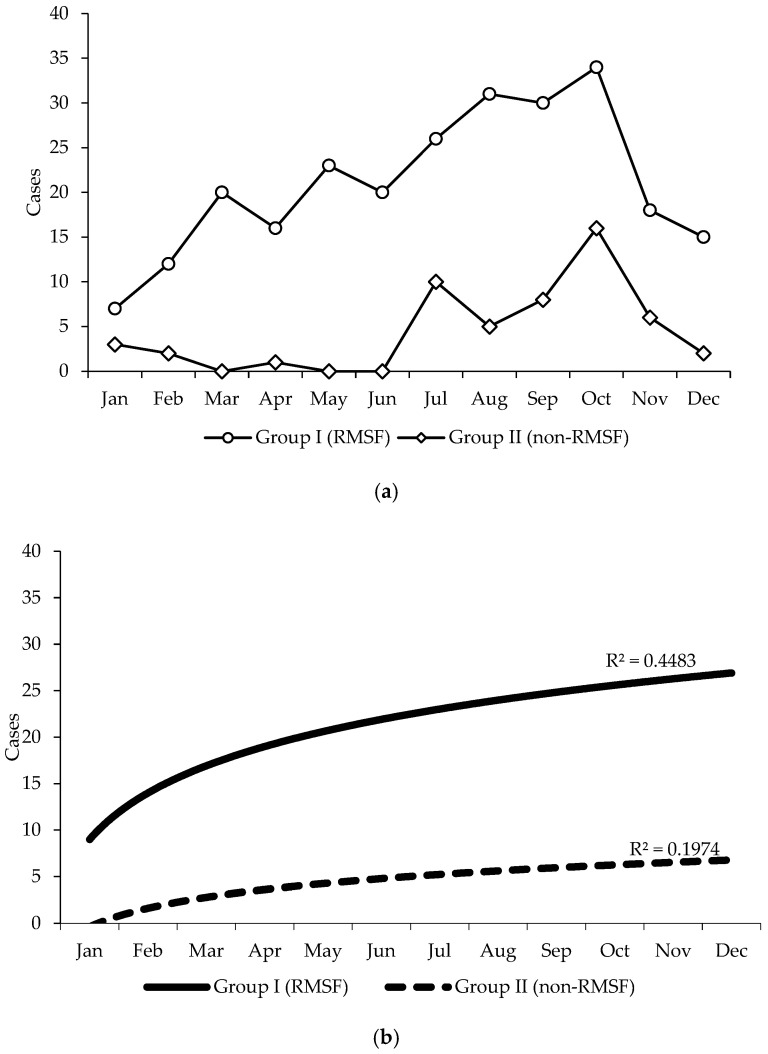
(**a**) Comparison of the occurrence of hospitalized pediatric cases of RMSF (n = 252) and non-RMSF (n = 53), according to month. Sonora, Mexico, 2015–2022. (**b**) Epidemiological trend in hospitalized pediatric RMSF (n = 252) and non-RMSF (n = 53) patients, according to month of occurrence. Sonora, Mexico, 2015–2022.

Although cases usually occur throughout the year, there was an epidemic peak across July–October, which is the warmest and most humid season across the state (Figure 2a). Even though both groups showed a growing tendency over the year, it was most notable in the RMSF group (R^2^ = 0.44) than in group II (R^2^ = 0.19), and such a difference was significant (*p* ≤ 0.005) (Figure 2b).

Regarding clinical manifestations, fever, headache, and maculopapular rash were the most frequently reported at the time of hospitalization for both groups, although they were consistently higher in group I. In fact, it was found that fever (RR = 1.25, CI 95% 1.11, 1.40) and maculopapular rash (RR = 2.7, CI 1.87, 3.98) were significantly higher in patients with RMSF. Likewise, petechiae (RR = 5.2, CI 95% 2.59–10.41) was more frequent in group I than in group II, which was marked when it involved palms (RR = 19.9, CI 95% 5.11–77.82) or soles (RR = 38.2, IC 95% 5.47–266.67). Overall, the rest of clinical manifestations were more frequent in group I than in group II (Table 2).

On the other hand, although a decrease in the platelet count was observed for both groups, the mean count was lower (59.75 × 10^3^/μL) in patients with RMSF than those from group II (110.00 × 10^3^/μL), (*p* < 0.001). Interestingly, the mean serum value of sodium (131.49 mEq/L) was lower in group I than in group II (136.08 mEq/L) (*p* ≤ 0.001). Moreover, the prothrombin time (15.68 s) and thromboplastin partial time (40.07 s) were both more elongated in patients with RMSF, but only the latter had significant difference (*p* < 0.001). Furthermore, the mean serum values of procalcitonin (4.33 ng/mL), ferritin (1081.24 ng/mL), and lactate dehydrogenase (752.97 U/L) were higher in group I than in group II, and all these differences were statistically significant. Although we did not observe statistical differences, the values of C-protein (11.98 mg/mL) and D-dimer (5.11 µg/mL) were higher in patients from group I (Table 3).

Regarding variables related to medical care, there was no difference (*p* = 0.583) in the days since the clinical onset of illness and the first medical attention (median = 3 days) for both groups. Nonetheless, patients of group I (median = 5 days) were hospitalized two days later than patients from group II (*p* = 0.002); patients with RMSF had three more days (median = 10 days) of hospital time than the dengue/COVID-19 group (*p* = 0.002). Furthermore, the total CFR (21.0%) in this series of patients with RMSF was significantly higher (*p* = 0.005) than for group II. The period presented 35 deaths in group I but only one occurred in patients in group II; in addition, 19 patients within group I were discharged with clinical sequelae (Table 4).

## 4. Discussion

Our findings show that children and adolescents hospitalized for RMSF present clinical and epidemiological features that can distinguish them from pediatric patients hospitalized either by dengue or COVID-19. At the first evaluation in the emergency room, clinicians can be guided toward early suspicion of RMSF by identifying a set of clinical manifestations—such as fever, maculopapular rash, thrombocytopenia, and hyponatremia—combined with a positive history of tick exposure even in absence of documented tick bite. This approach can guide clinicians to differentiate RMSF from dengue and COVID-19, even in endemic regions where all three diseases may co-occur year-round [5,24,25]. In these regions, early and empirical treatment with doxycycline for RMSF should be initiated, even in the absence of laboratory confirmation of *Rickettsia rickettsii* infection.

It should also be considered that there is an overlap in the incubation periods of RMSF (5 days) [6], dengue (5.6 days) [26], and COVID-19 (6.9 days) [27], which poses a challenge for physicians. The superposition of clinical manifestations observed can be explained by shared pathophysiological mechanisms among these diseases, primarily endothelial damage, procoagulant status, and proinflammatory response involving the release of cytokines such as interleukins (ILs) 1 and 6 or tumor necrosis factor-alpha (TNF-a). Such mechanisms can complicate clinical diagnosis and misguide the specific management of these diseases [18,19,20]. Despite the clinical similarities present in the three conditions, remarkable differences in the rapid progression of RMSF often lead to divergent clinical manifestations and outcomes compared with dengue and COVID-19, as well as other acute febrile undifferentiated illness, including malaria, leptospirosis, and spotted fever group rickettsiosis [28].

In the early stages of these diseases, fever, myalgia, arthralgia, and rash are clinical features that may overlap, differing primarily in their frequency of occurrence [13,29,30,31]. A careful examination of the rash can help clinicians distinguish between these diseases. In the early stages, RMSF patients develop a faint rash that typically appears 2 to 4 days after the onset of symptoms. Notably, fewer than 50% of patients present with a rash during the first three days of illness, and it may be absent in up to 10% of cases. This rash, consisting of 1–5 mm blanching macules, initially appears on the wrists, forearms, or ankles and spreads centripetally to the limbs and trunk. As the disease progresses, the rash may become petechial, indicating advanced microvascular injury, and it can involve the palms and soles [6,32]. In contrast, dengue fever typically presents with a small maculopapular rash on an erythematous background, resembling sunburn and feeling slightly rough to the touch. Some patients may develop faint petechial streaks in the axillary, antecubital, and inguinal areas. Petechiae may occur in severely ill patients; however, they usually do not involve the palms or soles [33,34]. Dermatological manifestations of COVID-19 may include a mild maculopapular rash, erythematous eruptions, and an itchy rash [35].

In addition to clinical examination, physicians should utilize, depending on availability, rapid antigen-based tests or RT-PCR for diagnosing either dengue [36] or COVID-19 [37], alongside epidemiological and social determinants to accurately suspect and guide therapeutic management. While RMSF requires the timely administration of doxycycline, the drug of choice, either orally or intravenously at doses of 200 mg for children weighing > 45 kg or 2.2 mg/kg per dose twice daily for children ≤ 45 kg [6]. Critical RMSF cases additionally can require fluid repletion, careful monitoring of fluid status, ventilatory mechanical support, dialysis, and other critical supportive interventions [38,39]. On the other hand, dengue is generally a self-limiting disease, managed symptomatically with rest and fluid electrolyte replacement [40]. In contrast, COVID-19 may be treated with antiviral agents targeting the host [41].

Remarkably, a proportion of patients of RMSF and dengue progress to severe manifestations such as petechial rash, hypotension, edema, septicemia, and multiple organic failure, highlighting the need for critical management, as recommended by international health agencies [6,36]. We emphasize that these severe manifestations are delayed and should neither be used for the early suspicion of RMSF nor for differentiating it from dengue, COVID-19, or other infectious diseases such as leptospirosis, meningococcemia, and other viral exanthematic childhood diseases. Notably, none of these diseases were reported in the state of Sonora during the analyzed period. Considering the nonspecific set of symptoms at the early stages of all three diseases, primary care physicians in endemic regions should carefully consider all three conditions in their differential diagnosis repertoire.

Consistent with previous studies [11,12,13,42], RMSF patients in our series showed a significant thrombocytopenia (mean value = 59.75 per 10^3^/μL) and hyponatremia (mean value = 131.49 mEq/L), both statistically different in patients from group II. Platelet counts below 100 per 10^3^/μL often occur in dengue; however, they are usually below 50 per 10^3^/μL in hospitalized patients with RMSF [42]. Nevertheless, when we excluded COVID-19 patients, thrombocytopenia was no longer different, which may be explained because the low platelet count is a rare event (8–9%) in hospitalized children with severe forms of COVID-19, including multisystemic inflammatory syndrome (MIS-C) [43,44].

Since some pathophysiological mechanisms are similar and underlie the clinical progression and fatal outcomes in patients hospitalized for any of the three conditions studied, in endemic regions, it is essential to routinely assess severity-related serological markers. Previous reports [3,31,44,45,46,47] have pointed out an increase in proinflammatory markers such as C-reactive protein, ferritin, and D-dimer for all three diseases. These markers can be valuable as severity predictors. Nonetheless, they are not usually measured in RMSF but only when COVID-19 is suspected, restraining the probability to predict the development of severe clinical manifestations and fatal outcomes in infected patients by *R. rickettsii*.

We recognize the challenges clinicians face when attempting to establish an accurate differential diagnosis at the early stages of these diseases. The routine use of epidemiological clues can overcome the limitations imposed by the clinical similarities of these three conditions, particularly in regions where RMSF, dengue, and COVID-19 are endemic. A thorough inquiry into the patient’s history of tick exposure, contact with animal hosts either domestic (e.g., dogs) or wild, and socioenvironmental conditions such as a high density of free-roaming dogs, poor health infrastructure, and poverty rates are essential. In Mexico and some endemic regions in the US, tick exposure and social disadvantage are common in patients with RMSF [1,5,13,48], which aligns with our findings. While dengue [49] and COVID-19 [50] can also affect underprivileged individuals and communities, the history of exposure to ticks remains as a hallmark of RMSF and can guide pediatricians to timely suspect the disease in any patient with febrile rash, even at the early clinical stage [5,42].

In the study region, we observed all three diseases occur year-round, although RMSF showed a seasonal peak during the July–October period, likely related to elevated environmental temperature (25–37 °C) and humidity (45–51%) that favor the host–tick interaction. It should be emphasized that this seasonal peak of RMSF does not rule out the presence of cases and outbreaks in any season. Frontline physicians should be aware about RMSF all year to overcome clinical dilemmas leading to misdiagnosis [51], particularly because there is regional evidence of coinfection between *Rickettsia rickettsii* and dengue virus, as well as other arboviruses [52,53] and COVID-19 [54].

Our findings confirm that RMSF, when not appropriately suspected and treated, is one of the most lethal acute infectious diseases in the pediatric population, with a CFR significantly higher than that of dengue [55] and COVID-19 [30,43]. The high CFR (21%) we observed underscores the importance of raising physician awareness to improve early suspicion of RMSF and prevent fatal outcomes, which may be underestimated when only deaths are considered as fatality. Most reports of fatality in RMSF are based exclusively on death, without addressing the long-term or permanent sequelae, such as amputations or neuromuscular, cardiac, and respiratory complications [56,57,58]. To better estimate the true burden of RMSF, fatal outcomes should systematically include both deaths and long-term sequelae.

Our study has several limitations. Firstly, we are not able to establish causal inferences because of the chosen study design. The retrospective data collection based on medical charts makes our findings prone to information bias. There was also potential selection bias due to the absence of COVID-19 cases during the 2015–2019 period. In addition, there was potential misclassification because the laboratory protocols to confirm cases varied over the study period.

## 5. Conclusions

Although RMSF, dengue, and COVID-19 can display some clinical and epidemiological similarities, mainly the presence of fever, malaise, and headache, they are more common in RMSF pediatric patients, which in our series, were admitted at their fifth day of clinical evolution. Such a delay can explain the differences we observed in the clinical profile of RMSF when compared either to dengue or COVID-19, the more prominent difference being the presence of petechial rash, thrombocytopenia, and hyponatremia, all reflecting vascular injury and increased vascular permeability. None of these manifestations should be used for the early suspicion of any of the diseases we document. We emphasize the need to strengthen clinician training to routinely integrate epidemiological clues into the diagnosis of febrile illnesses in regions where RMSF, dengue, and COVID-19 overlap. Remarkably, a recent history of tick contact is a distinctive feature that can guide medical decisions in clinical scenarios. In regions where RMSF, dengue, and COVID-19 are endemic, physicians should be aware that RMSF provokes higher numbers of fatality than the other two diseases, thus requiring early suspicion and immediate specific treatment.

## Figures and Tables

**Table 1:** Selected characteristics of study subjects. Sonora, Mexico, 2015–2022.

Variable	N (%)		*p*-Value ^1/^
	Group I (n = 252)	Group II (n = 53)	
Method of confirmation			
RT-PCR	98 (38.9)	19 (35.8)	<0.001
Antibody/antigen detection ^2/^	52 (20.6)	34 (64.2)	
Clinical epidemiological	102 (40.5)	0 (0)	
Sex			
Male	135 (53.6)	23 (43.4)	0.178
Female	117 (46.4)	30 (56.6)	
Age (mean ± SD)	8.86 ± 4.29	8.21 ± 6.55	0.489 *
Grouped age			
0–4	45 (17.9)	23 (43.4)	<0.001
5–9	105 (41.7)	2 (3.8)	
10–14	76 (30.2)	18 (34.0)	
15–19	26 (10.3)	10 (18.9)	
Belongs to an ethnic group			
Yes	11 (4.4)	0 (0)	0.388
No	241 (95.6)	53 (100)	
Socioeconomic status			
Very low	96 (38.1)	12 (22.6)	
Low	103 (40.9)	21 (39.6)	0.018
Intermediate	53 (21.0)	20 (37.8)	
Type of locality ^3/^			
Urban	228 (90.5)	43 (81.1)	0.049
Rural	24 (9.5)	10 (18.9)	
Region of residence ^4/^			
North	28 (11.1)	3 (5.7)	0.410
Center	185 (74.4)	43 (81.1)	
South	39 (15.5)	7 (13.2)	
History of tick exposure			
Positive	232 (92.1)	5 (9.4)	<0.001
Negative	20 (7.9)	46 (86.8)	
Unknown	0 (0)	2 (3.8)	

^1/^ Based on χ^2^ test. * Based on Student’s *t*-test. PCR-tr: real-time polymerase chain reaction. ^2/^ Group I used IFA; in group II, ELISA or NS1 antigen detection were used. ^3/^ Urban refers to a locality > 15,000 inhabitants. Rural is a locality < 15,000 inhabitants. ^4/^ North region includes the health districts (HDs) of Caborca, Santa Ana, and San Luis Río Colorado; center includes HDs of Hermosillo; south includes HDs of Ciudad Obregón and Navojoa.

**Table 2:** Comparison of clinical characteristics in study subjects. Sonora, Mexico, 2015–2022.

Characteristic	N (%)		Risk Ratio (95% CI) ^1/^
	Group I (n = 252)	Group II (n = 53)	
Fever (self-reported)	249 (99.0)	45 (79.2)	1.25 (1.11, 1.40)
Maculopapular rash	234 (92.9)	18 (34.0)	2.73 (1.87, 3.98)
Headache	224 (88.9)	40 (75.5)	1.18 (1.00, 1.38)
Petechial rash			
- Palms	190 (75.4)	2 (3.78)	19.95 (5.11, 77.82)
- Soles	183 (72.6)	1 (1.9)	38.21 (5.47, 266.67)
- Generalized	173 (68.6)	7 (13.2)	5.20 (2.59, 10.41)
Myalgias	184 (73.0)	17 (32.1)	2.27 (1.52, 3.38)
Arthralgias	172 (68.2)	19 (35.8)	1.91 (1.31, 2.75)
Hypotension	152 (60.3)	5 (9.4)	6.41 (2.76, 14.86)
Abdominal pain	132 (52.4)	16 (30.2)	1.74 (1.13, 2.65)
Vomiting	126 (50.0)	19 (35.8)	1.40 (0.95, 2.04)
Confusion	90 (35.7)	6 (11.5)	3.10 (1.43, 6.69)
Ankle edema	90 (35.7)	1 (1.9)	18.79 (2.67, 131.86)
Wrist edema	73 (29.0)	1 (1.9)	15.26 (2.16, 107.38)
Hepatomegaly	56 (22.2)	3 (5.7)	3.89 (1.26, 11.97)
Conjunctivitis	40 (15.9)	2 (3.8)	4.18 (2.41, 7.26)
Renal failure	29 (11.5)	1 (1.9)	6.05 (0.84, 43.46)

^1/^ Group II (non-RMSF) was used as the reference group. 95% CI: 95% confidence interval.

**Table 3:** Comparison of biomarkers at hospital admission in study subjects. Sonora, Mexico, 2015–2022.

Variable (Reference Value)	Geometric Mean (N)		*p* Value ^1/^
	Group I (n = 252)	Group II (n = 53)	
Hemoglobin (12.2–18.1 g/dL)	11.67 (249)	12.87 (51)	0.001
Leukocytes (4.6–10.2 × 10³/μL)	8.53 (251)	6.49 (51)	0.050
Platelet count (150–450 × 10³/μL)	59.75 (250)	110 (50)	<0.001
Prothrombin time (11.1–14.1 s)	15.68 (225)	14.93 (30)	0.140
Partial thromboplastin Time (20–40 s)	40.07 (225)	35.27 (30)	<0.001
Procalcitonin (0–0.5 ng/mL)	4.33 (151)	0.27 (19)	<0.001
C-reactive protein (<0.5 mg/mL)	11.98 (22)	4.92 (5)	0.720
D-dimer (0–0.5 µg/mL)	5.11 (12)	4.85 (5)	0.420
Ferritin (21–274 ng/mL)	1081.24 (14)	578.45 (2)	0.031
Lactate dehydrogenase (240–480 U/L)	752.97 (196)	522.82 (29)	0.008
AST (5–34 U/L)	103.65 (238)	77.20 (37)	0.120
ALT (0–55 U/L)	51.42 (233)	36.33 (37)	0.340
Serum sodium (136.0–146.0 mEq/L)	131.48 (244)	136.08 (46)	<0.001

AST: aspartate aminotransferase; ALT: alanine aminotransferase. ^1/^ Based on a *t*-test for independent samples.

**Table 4:** Medical attention and outcomes in study subjects. Sonora, Mexico, 2015–2022.

Variable	Median (IQR) [n]		*p* Value ^1/^
	Group I (n = 252)	Group II (n = 53)	
Days from onset of symptoms to			
(a) First medical attention	3.00 (1.00–4.75) (206)	3.00 (1.00–4.00) (46)	0.583
(b) Hospital admission	5.00 (3.00–6.00) (252)	3.00 (1.00–6.00) (53)	0.002
(c) Hospital discharge	10.00 (7.00–16.00) (252)	7.00 (4.5–11.5) (53)	0.002
Deaths [N (%)]	34 (13.49)	1 (1.88)	0.005 *
Sequelae at discharge [N (%)]	19 (8.72)	1 (1.92)	

^¹/^ Based on the Mann–Whitney U test. * Based on a chi-squared test with Yates’ correction. IQR: interquartile range.

## Data Availability

Data used for conducting this research are not publicly available due to privacy and ethical restrictions.
